# Supplementary figures and images for: Inheritance-Specific Dysregulation of Th1- and Th17-Associated Cytokines in Alopecia Areata
Source: Biomolecules. 2023 Aug 23;13(9):1285. doi: 10.3390/biom13091285 (PMC10527519; doi:10.3390/biom13091285)

## Supplemental Figure S1.

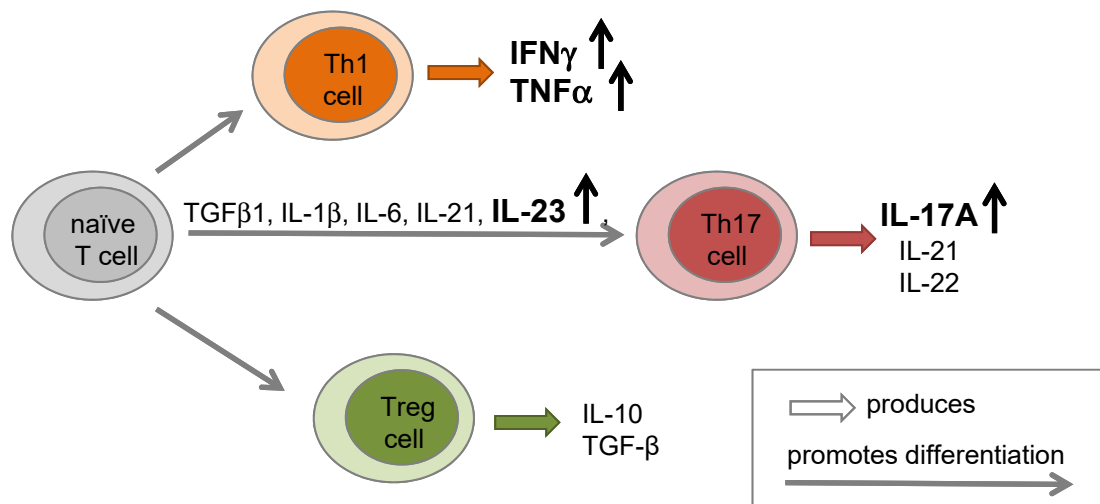

Supplement: Supplementary file 1 [file biomolecules-13-01285-s001.zip › biomolecules-2527735-supplementary.pdf]
